# Tailoring ultrabroadband near‐infrared luminescence in Bi-doped germanosilicate glasses

**DOI:** 10.1038/s41598-023-49898-1

**Published:** 2023-12-21

**Authors:** A. Mehaboob, V. Fuertes, V. A. G. Rivera, Y. Messaddeq

**Affiliations:** Centre d’optique, Photonique et Laser, Université Laval, 2375 Rue de la Terrasse, Quebec, QC G1V 0A6 Canada

**Keywords:** Optics and photonics, Fibre optics and optical communications, Glasses, Materials for optics

## Abstract

Bi-doped glasses and optical fibers are extensively studied since they present broadband optical amplification in the near-infrared region (NIR), in which the optical telecommunication industry greatly depends for the transmission of optical signals. There are many scientific challenges about the NIR luminescent emissions from Bi ions, such as understanding its origin and further improving the associated optical amplification capacity. In this work, Bi-doped germanosilicate glass compositions with ultrabroadband NIR luminescence were fabricated, in the range of 925–1630 nm, which covers O, E, S, C, and L-telecommunication bands. An in-depth analysis of the impact of modifying excitation wavelengths, Bi content, and GeO_2_/SiO_2_ concentration ratio in the glass matrix demonstrates the possibility of considerably manipulating the Bi NIR luminescence, in terms of tuning emission parameters such as bandwidth, up to ~ 490 nm, and luminescence intensity. Based on theoretical and experimental luminescence data retrieved from the fabricated glasses, we demonstrate that the origin of broadband luminescence under all the considered excitation wavelengths can be ascribed to optical transitions of Bi^0^ ions. Therefore, an energy level diagram for Bi^0^ is proposed. We anticipate that our findings can provide clarifications to the existing uncertainty in the origin of Bi NIR emission, which will be useful to fabricate efficient future optical fiber amplifiers.

## Introduction

The current data rate transmission capacity has already exceeded several Tbit/s, and in the upcoming years, transmission in the range of Pbit/s will need to be reached to compete with the continuous increasing demand for internet and data transfer^1,2^. At present, the optical fiber-based technology is mainly concentrated in the C- and L- bands in which the Wavelength Division Multiplexing (WDM) along with Erbium Doped Fiber Amplifier (EDFA) provide an excellent performance to maximize the use of this wavelength region^3^. Furthermore, active researches are ongoing for extending the amplification region of EDFA beyond the L-band^4^. Despite of this success, currently, the EDFA presents the drawback that it only covers 80 nm of the low loss region of silica fibers extended from 1300 to 1700 nm for the practical applications, which limits its applicability^5^. Thus, the discovery of novel glasses for the fabrication of optical amplifiers suitable for this entire low-loss region of silica fibers will have a remarkable impact on the future of the telecommunication industry.

Novel potential materials that might improve the performance of EDFA-based technology are explored. In this regard, Bismuth-doped glasses (BDGs) and Bismuth-doped optical fibers (BDOF) have recently received large attention owing to their broad luminescence in the NIR region, ranging from 1000 to 1800 nm, depending on the host matrix considered and the pumping wavelength^6,7^. This feature makes them unique regarding the rare-earth ions (REI) analogues. However, even two decades after Fujimoto et al.^8^ proposed Bismuth (Bi^x^, where x is the oxidation state) as a potential candidate for optical amplifiers, its origin and the luminescent mechanism still remains uncertain. The main incertitude regarding the origin of the NIR luminescence arises from the fact that unlike the well shielded 4f shells of REI, the valence electrons in 6s and 6p outer electronic shells of Bi^x^ are barely shielded. Thus, Bi^x^ ions are sensitive to the host glass composition and synthesis environment^9^. Hence, not only the effect of the crystalline field should be considered here, but also the spin–orbit interaction, Coulomb repulsion, and the effective Coulomb potential^10^. Furthermore, Bi^x^ ions can adopt multiple oxidation states^11^, and those polyvalent ions are in reduction/oxidation (redox) equilibrium in molten glass. Thus, Bi^x^ oxidation state may change at a rapid rate during the fabrication process, both melting and for annealing, decreasing its valence state as fabrication temperature increases, which hinders its control throughout the fabrication process^1,5^. Even though numerous studies have identified various Bi^x^ ions as potential sources of NIR luminescence^9^, a detailed investigation that incorporates both experimental and theoretical atomic spectral data as well as the different factors that affect the nature of optical transitions is not reported according to our best knowledge, particularly for Bi^x^- doped germanosilicate glasses.

It is well known that germanosilicate glasses combine the benefits of both silicate and germanate glasses, such as high refractive index, high thermal stability, as well as lower phonon energy comparing to silicate glasses^12^. Recently, Bi^x^-doped germanosilicate glasses and optical fibers have received great deal of attention due to their ability to present NIR emissions in O-, E-, S-, C-, L- and U- telecommunication bands, which is not possible in Er^3+^- doped glasses^2,5,13^. There are some works that report the possibility of manipulating NIR emissions in BDOFs by changing the silica-based host composition. In this context, fiber core compositions made of a germanosilicate host were reported to exhibit promising luminescence performance in E-, S-, L- and U- telecommunication bands depending on the amount of GeO_2_¸incorporated in the core^2,14,15^. However, in bulk glasses, a broad emission in the whole NIR range of 1000–1600 nm was observed for Bi^x^-doped glasses in the system SiO_2_–Al_2_O_3_^8^, germanate^16^, and glasses with no Ge or Si content in their compositions such as aluminophosphate glasses^17^. This evidence shows that NIR luminescence from Bi^x^ ions is a complex phenomenon that remains unclear, and there is still a need for a detailed investigation, mainly from a material point of view, to evaluate the effect of host glass composition. Therefore, in the case of germanosilicates, which have shown high potential for fiber amplifier applications^18^, a systematic study about the impact of changing the GeO_2_/SiO_2_ concentration ratio on NIR luminescence will help to analyze the role of both Si and Ge, while keeping other elements constant. This will help to understand the structural evolution occurring in the glasses and which element (Si or Ge) strongly influences NIR luminescence. However, to date, according to our best knowledge, this investigation has not been carried out for germanosilicate glass compositions similar to the ones discussed in this work.

In this context, the current work aims to fabricate tunable ultrabroadband NIR-emitting Bi^x^-doped germanosilicate glasses, with a NIR luminescence that can be tailored in the O-, E-, S-, C- and L- telecommunication bands. A systematic study about the effect of both SiO_2_ and GeO_2_ on the glass matrix and luminescence, as well as the effect of changing Bi^x^ concentration and excitation wavelengths (λ_ex_) is considered, while keeping other fabrication parameters constant. The possible origin of Bi^x^ NIR emission is thoroughly discussed with support of both theoretical and experimental data. The extracted conclusions contribute to the understanding about luminescence of Bi^x^-doped germanosilicate glasses, which will be helpful for future optical fiber amplifiers based on Bi^x^.

## Experimental methods

### Glass fabrication

**Figure 1:**
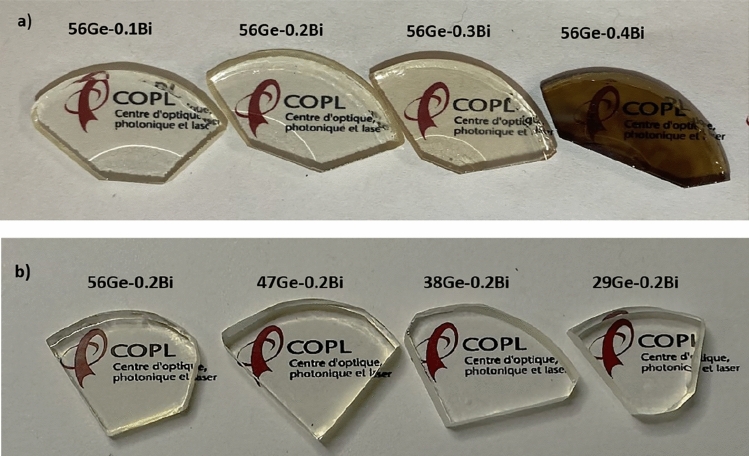
Glass samples in the series: (**a**) 56Ge-xBi and (**b**) (56 − x) Ge–0.2Bi. All glasses were annealed at 620 °C for 5 h with an average thickness of 3 mm.

Glass compositions were fabricated in two sets of series: (i) (100 − x) (56 GeO_2_–18 SiO_2_–23 BaO–3Al_2_O_3_) xBi_2_O_3_ where x = 0, 0.1, 0.2, 0.3 and 0.4 mol% (labeled as 56Ge-xBi) and ii) (56 − x) GeO_2_–(18 + x) SiO_2_–23 BaO–3Al_2_O_3 _− 0.2 Bi_2_O_3_ where x = 0, 9, 18 and 27 mol% (labeled as (56 − x) Ge–0.2Bi. High purity reagents were used such as GeO_2_(5N), SiO_2_ (5N), BaO (4N), Al_2_O_3_(6N), and Bi_2_O_3_ (3N). To reduce the influence of Al_2_O_3_ and BaO on Bi^x^ luminescence and isolate as much as possible the role of GeO_2_, their concentration was optimized and tried to be kept in the lowest amount possible for all glasses, within the experimental limitations related to the highest temperature that the melting furnace used could achieve. Initially, the precursors are crushed and mixed for 10 min by using a mortar and pestle to obtain a homogenous powder. Then, the mixture of reagents was placed in a platinum crucible and melted at a temperature of 1650 °C for 1 h in air by using a melting furnace. Subsequently, the glasses were quenched to room temperature while keeping the melt in the crucible and the resulting glasses were extracted from the crucible. Then, the glasses were annealed at a temperature of 620 °C for 5 h after extraction. Finally, the annealed samples were polished to an average thickness of 3 mm to carry out the optical characterizations and allow their comparison (Fig. 1).

### Characterization

Thermal characterization was performed by differential scanning calorimetry (DSC) method to measure the characteristic temperatures such as T_g_ (glass transition temperature), T_x_ (onset of crystallization temperature) and T_p_ (peak crystallization temperature). For this purpose, a Netzsch DSC Pegasus 404F3 instrumental setup was used. The structural features of the glasses were analyzed by Raman spectroscopy. Spectra were recorded using a Renishaw in via spectrometer coupled to a Leica DM2700 microscope in the spectral range 100–1200 cm^−1^ and using λ_ex_ of 633 nm. With respect to optical characterization, the refractive index of the glass samples was determined at the operating wavelengths of 532, 633, 972, 1308 and 1538 nm by using the prism coupling technique (Metricon Model 2010/M Prism Coupler). Luminescence spectra for the glass samples were recorded by a Nanolog spectrofluorimeter from Horiba Jobin Yvon equipped with PMT-NIR detector which can be switched to a photomultiplier Hamamatsu NIR-PMT module detector. The samples were pumped under λ_ex_ of 377, 464 and 824 nm. The spectral slit width was 3 nm for emission and the acquired data were corrected by instrumental factors. For the lifetime decay measurements, an OPO centered at 464 and 824 nm was used as the pump source and an Edinburgh FLS1000 spectrometer coupled with a Tektronix oscilloscope to record the time-dependent decays. The lifetime measurement was only carried out for λ_ex_ of 464 and 824 nm and not for 377 nm, due to the limited operational wavelength range of OPO laser (410–2400 nm). For the luminescence measurements, the samples were put in the same position as the others to ensure good comparability between the luminescence spectra. All the optical measurements above mentioned were made at room temperature.

## Results and discussion

The fabricated germanosilicate glasses were transparent and without bubbles, as shown in Fig. 1. The glasses with different concentrations of Bi_2_O_3_ were fabricated to examine the impact of varying Bismuth content and to find an appropriate Bi_2_O_3_ nominal concentration, for later, carrying out systematic research with different GeO_2_ concentrations. The aim of varying the concentration of GeO_2_ was to investigate its influence on both the structure and luminescence of the fabricated Bi^x^-doped germanosilicate glasses with the aim of tailoring the associated NIR emission. Considering this, the glass with 0.2 mol % of Bi_2_O_3_ was selected due to the fact that this glass shown no sign of reduction after annealing (Fig. 1a). As seen in Fig. 1a, glasses above 0.2 mol% of Bi_2_O_3_ displayed a change of color after annealing treatments, turning brownish, which is generally related to the reduction of Bismuth ions into metallic Bi (Bi^o^) state and subsequent migration and aggregation of Bi^o^ ions to form Bi nanoparticles^19^. Several works reported a decrease in luminescence when Bi^x^ is reduced to lower valence states^19,20^. The discussions drawn from the different characterization techniques used are provided hereafter.

### Thermal characterization: DSC

**Figure 2:**
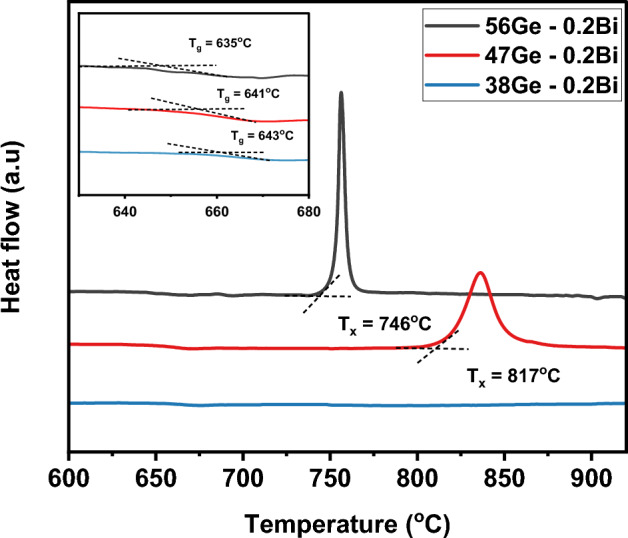
DSC curves measured for the glasses in the series (56 − x) Ge–0.2Bi.

DSC spectra measured for the glasses in the series (56 − x) Ge–0.2Bi are shown in Fig. 2, and the corresponding thermal parameters such as T_g,_ T_x_ and T_p_ are depicted in the graph along with an enlargement of glass transition region, displayed as inset, for the three samples. The value of T_g_ and T_x_ obtained from the DSC spectra and along with the thermal stability parameter ΔT (= T_x_ − T_g_) were provided in Table S1 of supporting information. The parameter ΔT provides a direct measure of resistance to nucleation and crystallization^21^. Glasses with larger ΔT exhibits strong inhibition to nucleation and crystallization. From Fig. 2, it is noticed that the thermal stability of the samples increases with decrease in GeO_2_ content. A decrease of 9 mol % of GeO_2_ considerably increases ΔT, from 111 °C for the sample 56Ge–0.2Bi to 176 °C for the 47Ge-0.2Bi sample (Table S1). In light of the work reported by Wang et al. the thermal stability of germanate glasses improves by the addition of SiO_2_ into the glass network since silica can enhance the glass network strength by connecting it through bridging oxygens (BO), and therefore make it more thermally stable^22^. The T_g_ values could be extracted only up to the sample 38Ge–0.2Bi, since the characteristic transitions were not fully observed in the DSC of the two samples 38Ge–0.2Bi and 29Ge–0.2Bi. However, according to the behavior observed here, an increase of thermal stability with respect to 38Ge–0.2Bi sample is expected. As previously mentioned in glass fabrication section, an annealing temperature of 620 °C was selected for all the glasses based on the above discussion.

### Structural characterization—Raman spectroscopy

**Figure 3:**
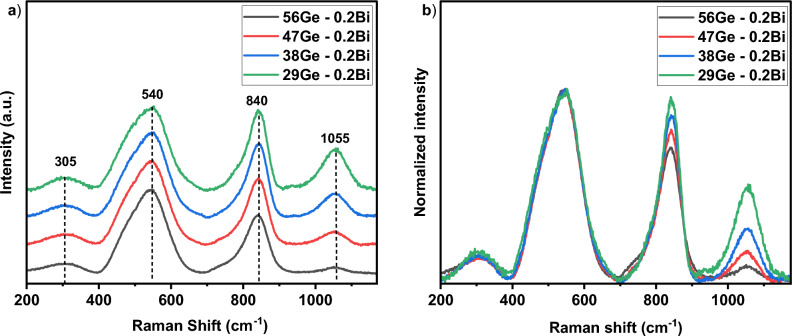
(**a**) Raman spectra measured for the glasses in the series (56-x) Ge–0.2Bi and **b)** normalized Raman spectra for the same series.

Figure 3a shows the Raman spectra for the glasses in the series (56 − x) Ge–0.2Bi, in which the effect of decreasing GeO_2_ concentration on the vibrational behavior was observed. All spectra consist of four main peaks located at 305, 540, 840 and 1055 cm^−1^ as well as a small shoulder band at 770 cm^−1^. The first peak at 305 cm^−1^ is related to the bending modes of Ge–O–Ge bonds, which is derived from stretching vibrations of GeO_6_ connected units^23,24^. Several studies reported in the ^23,25^ suggest that although this band is located at $\sim$ 343 cm^−1^ in pure GeO_2_ glasses, it can be shifted to lower frequencies by adding cation modifiers such as Na, Cs, or K to germanate glasses or by adding Ga to alkali gallogermanate glasses. In the current work, this behavior is also observed and can be ascribed to the presence of high content of Ba, 23 mol%, which acts as a glass network modifier. The broad peak at 540 cm^−1^ is due to the symmetrical stretching modes of BOs in T–O–T bonds (where T = Ge/Si) of either Ge or Si tetrahedra^26,27^. The small shoulder band at 770 cm^−1^ can be assigned to both the movement of Si atoms in its oxygen network and additionally the vibration of oxygen atoms in the Ge tetrahedra^28^. This band is less noticeable as GeO_2_ amount is reduced. The band peaked at 840 cm^−1^ is linked to the stretching mode of Si–O–Si while the wide band centered at around 1055 cm^−1^ is assigned to the asymmetric stretching modes of Si–O–Si bonds in Q^4^ tetrahedral units of SiO_4_^26,27,29^. The normalized spectra for the samples are plotted in Fig. 3b. In the high frequency region, the two main bands at 840 and 1055 cm^−1^ tend to become more intense and wider as GeO_2_ content decreases. This is attributed to the fact that when the concentration of GeO_2_ decreases, thereby SiO_2_ concentration increases, the population of SiO_4_ tetrahedral units increases in the glass system and thus, the Si–O–Si stretching mode becomes more prominent, in accordance with^30^.

These findings provide insight about the glass network in the system SiO_2_–GeO_2_–Al_2_O_3_–BaO–Bi_2_O_3,_ and evince that as GeO_2_ concentration decreases, a larger connectivity through BOs is present in the samples. No difference was noticed between the Raman spectra for the glasses in the series 56Ge–xBi (Fig. S1 of supporting information), and therefore no structural change occurs with the increase of Bi_2_O_3_ content, which is reasonable considering the low amount used and its role as dopant in the glass network.

### Optical characterization

#### Refractive index

**Figure 4:**
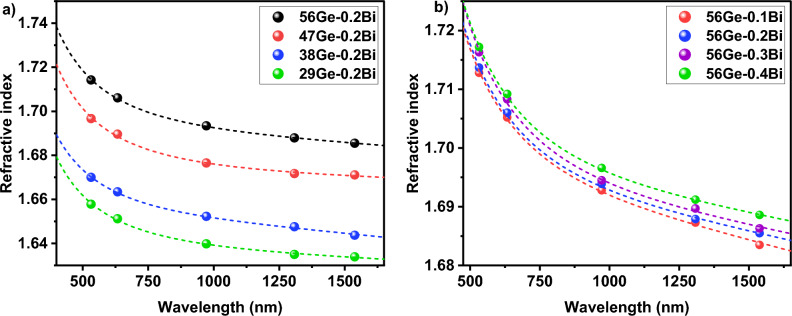
Refractive index of the glasses annealed at 620 °C for 5 h in the series: (**a**) (56 − x) Ge–0.2Bi and (**b**) 56Ge-xBi.

The refractive index measured for the glass samples in the series (56 − x) Ge–0.2Bi and 56Ge–xBi, both annealed for 5 h at 620 °C, is depicted in Fig. 4. As expected, the refractive index of the glasses varies with the wavelength due to chromatic dispersion phenomenon. Since refractive index is a function of electron density or electronic polarizability, the incorporation of highly polarizable ions or compositional changes that lead to an increase in concentration of highly polarizable non-bridging oxygens (NBO) can increase the refractive index of glasses^31,32^. From Fig. 4a, it is noticed that the refractive index of the glasses exhibits an increase with the increase in the GeO_2_ concentration, for a given wavelength. This can be explain by: (i) the replacement of SiO_2_ (n $\approx$ 1.470) by GeO_2_ (n $\approx$ 1.7), as glass former, since GeO_2_ has larger refractive index and larger polarizability; and (ii) the addition of GeO_2_ induce depolymerization of the silica network and thus the creation of more NBOs in the glass structure^33^. This observed behavior is in accordance with other studies^34,35^.

In the case of the glasses in the series 56Ge–xBi, the refractive index subtly increases as Bi_2_O_3_ content increases, in the whole spectra measured (Fig. 4b). This can be justified by the fact that Bi^x^ is an ion with large atomic number, presents high electron density, and also high refractive index, of ~ 2.5^31,36^. Furthermore, Bi_2_O_3_ can act as a glass modifier and its addition to the glass structure can provide a larger number of NBOs, by breaking BOs, and therefore increasing polarity and refractive index of the glasses^36^.

#### Luminescence measurements

**Figure 5:**
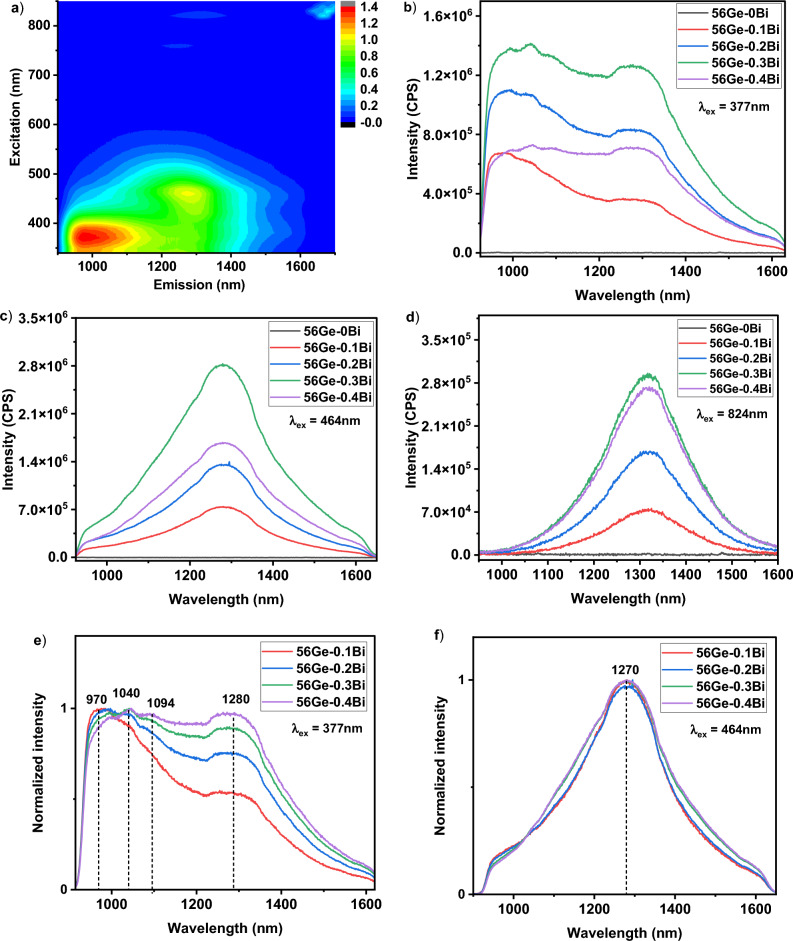
Tailoring luminescence of Bi^x^-doped germanosilicate glasses by modifying Bi dopant content. (**a**) Contour plot showing the emission-excitation wavelength dependence for the sample 56Ge–0.2Bi. Color code for intensity of emission is depicted in the scalebar; Luminescence spectra for samples annealed at 620 °C for 5 h in the series 56Ge–xBi under excitation wavelengths of (**b**) 377 nm (**c**) 464 nm and (**d**) 824 nm; Normalized luminescence spectra for (**e**) 377 nm and (**f**) 464 nm. The unit of luminescence intensity is given in CPS (counts per second).

The luminescence spectra measured for the samples 56Ge–xBi, annealed at 620 °C for 5 h, are displayed in Fig. 5. According to the data extracted from the emission-excitation plot recorded for the sample 56Ge–0.2Bi (Fig. 5a) as well as literature^19,37^, λ_ex_ of 377, 464 and 824 nm were selected. Figure 5a shows that our designed germanosilicate glasses present a tunable ultrabroadband NIR emission ranging from 925 to 1630 nm, by simply changing the λ_ex_, which may be associated to the presence of different Bi-related emission centers (BECs), as it will be discussed hereafter. A BEC is defined as a Bi^x^ ion that has a radiative emission that will depend on the environment around it, the local symmetry and its valence. It is important to mention that no emission was observed from the glass with 0 mol % of Bi_2_O_3_ under all three λ_ex_ considered, indicating that Bi^x^ ions are necessary for obtaining NIR emission in the fabricated germanosilicate compositions. Under λ_ex_ = 377 nm, all samples exhibited a ultrabroadband NIR luminescence in the range of 925–1630 nm (Fig. 5b). Furthermore, FWHM values in the range of 420–490 nm are observed (Table 1) and increase as the Bi_2_O_3_ nominal content increased. Moreover, as Bi_2_O_3_ concentration increases, the emission becomes more flattened and wider, covering O-, E-, S-, C- and L- telecommunication bands (See Fig. 5b). In particular, the sample 56Ge–0.4Bi exhibited a flattened emission, with a FWHM of around 490 nm that covers the NIR range from 925 to 1630 nm. This unique feature displayed by the sample 56Ge–0.4Bi in terms of emission flatness can be technologically very relevant in the field of optical fiber amplifiers.

**Table 1:** FWHM values for the fabricated glass samples in the series 56Ge–xBi.

λ_ex_ (nm)	FWHM (nm)			
	56Ge–0.1Bi	56Ge–0.2Bi	56Ge–0.3Bi	56Ge–0.4Bi
377	397	450	490	488
464	248	255	292	300
824	199	206	209	211

In the work reported by Yan et al. for Bi^x^-doped barium gallo-germanate (BGG) glasses^38^, the characteristics of the emission recorded under λ_ex_ = 350 nm is very different from the one observed in our current work. When excited at 350 nm, Yan et al. reported an NIR emission in the wavelength range of 900–1400 nm, which was peaked at 1120 nm and had a FWHM of 220 nm. However, in our work, an ultrawide emission in the 925–1630 nm range was obtained, with a maximum FWHM of nearly 490 nm for the sample 56Ge–0.4Bi (Table 1). Furthermore, Zhang et al. investigated the influence of Ge concentration on NIR luminescence of Bi^x^ in germanosilicate glasses^19^. The glasses displayed NIR emission with FWHM of about 300 nm at a wavelength of about 1260 nm for λ_ex_ = 808 nm. The optimum glass composition obtained was 13Li_2_O–23Al_2_O_3_–20GeO_2_–43SiO_2_–1Bi_2_O_3_^19^. In that case, the research was mainly focused on analyzing the emission-excitation spectra of the optimum developed composition and the most suitable λ_ex_ recorded was 470 nm. Furthermore, despite the fact that some research works suggested the possibility of using λ_ex_ = 377 nm in germanosilicate glasses, no extensive study has been carried out at this λ_ex_ to date^19^. However, for our glass system, we demonstrate that using λ_ex_ = 377 nm is optimum, in terms of emission intensity and width, which may be useful for future optical fibers.

Similarly, under the excitation at 464 nm, the samples exhibited a broad emission peaked at 1270 nm, in the wavelength range of 920–1650 nm. As for 464 nm, emission becomes more intense and wider as Bi concentration increases (Fig. 5c). In this case, the sample 56Ge-0.4Bi exhibited a maximum FWHM of around 300 nm while the sample 56Ge-0.1Bi showed a FWHM of 248 nm (Table 1). Cao et al. reported emission under 460 nm pumping, for a germano-phosphate glass host, and also demonstrated a FWHM around 300 nm, but using a larger Bi doping concentration, of around 1 mol %^37^. Furthermore, in Bi^x^-doped germanosilicate glasses, some research works reported emission under an excitation of around 470 nm^19,39^, but the observed emission peaks were either at 1165 nm or 1220 nm, which is different from the peak position observed at 1280 nm in the current work. This can be mainly attributed to the difference in the glass compositions used for these works. For example, Ren et al.^40^ reported that the use of various alkaline or alkaline earth oxides as glass modifiers had an impact on shifting the NIR luminescence peak in silicate glasses. In that work, the emission peak, which was originally measured at 1315 nm under λ_ex_ = 808 nm, underwent a red shift to 1325 nm and a blue shift to 1305 nm, respectively, when the initial alkali modifier BaO was replaced by CaO and SrO. The authors suggested that infrared luminescence is sensitive to the local environment of the BEC^40^.

With the aim of explaining this shift in the emission peak position, we consider a Hamiltonian that describes our electronic system as^10,41,42^:

1 $$\begin{aligned} H & = - \left( {H_{{\text{o}}} + H_{{\text{C}}} } \right) + H_{{\text{m}}} + H_{{{\text{SO}}}} + H_{{{\text{CF}}}} \\ & \quad = - \left( {\frac{{\hbar^{2} }}{2m}\mathop \sum \limits_{i = 1}^{N} \overrightarrow {{\nabla_{i} }}^{2} + \mathop \sum \limits_{i = 1}^{N} \frac{{{\text{Ze}}^{2} }}{{r_{i} }}} \right) + \mathop \sum \limits_{i < j}^{{N^{\prime}}} \frac{{e^{2} }}{{r_{ij} }} + \mathop \sum \limits_{i = 1}^{N} \xi \left( {r_{i} } \right)\overrightarrow {{S_{i} }} \cdot \overrightarrow {{L_{i} }} + V_{{{\text{CF}}}} \\ \end{aligned}$$

In this relation, the terms *H*_O_ represents the kinetic energy, *H*_C_ the effective Coulombian potential between the nucleus and the electrons in the central ion (in our case the central ion is Bi^x^), *H*_m_ describes the Coulombian repulsion between electrons from other atoms and central ion in the glass matrix,* H*_SO_ the spin–orbit interaction, and finally *H*_CF_ = $V_{{{\text{CF}}}}$, the crystal-field interaction taking place in an atomic system. Here, *N* is the number of electrons of Bi^x^ ion, *N’* is the number of electrons in the valence shell of Bi^x^, *Ze* is the effective charge, $\xi \left( {r_{i} } \right) \left( { = \frac{{Ze^{2} }}{{2m_{e}^{2} c^{2} r_{ij}^{3} }}} \right),$ $\vec{S}_{i}$ and $\vec{L}_{i}$ are the spin–orbit coupling efficiency, spin and the total orbital angular momentum, respectively, and *e* is the electron charge. The first two terms of Eq. 1 arise due to the Bi^x^ ion, while the other three terms depend on both the network symmetry and the network component ions, and they are responsible for the characteristic emission spectrum of the ion. The third term of the *H*_*m*_ gives the multielectron repulsion and it is the principal factor responsible for the energy shift of the maximum intensity peak on the emission spectrum. Additionally, the spin–orbit coupling, and crystal field interactions can modify the width of the emission spectrum. All three final terms depend on the atomic number and increase as atomic number increases. Therefore, the different line shapes, position peaks and broadband emissions from Bi^x^ ions reported in different works of literature can be explained with the Hamiltonian of Eq. 1.

With respect to λ_ex_ = 824 nm [See Fig. 5d], the glasses shown a luminescence in the range of 1000–1600 nm, peaked at 1320 nm and with a maximum FWHM around 220 nm for 56Ge–0.4Bi (see Fig. 5d and Table 1). Figure 5e and f depict the normalized spectra for λ_ex_ = 377 and 464 nm. No change with increasing Bi concentration was exhibited by the normalized spectrum for λ_ex_ = 824 nm, and therefore it is omitted. As previously mentioned, it is noticed a broadening of the emission for both λ_ex_ of 377 and 464 nm, as Bi_2_O_3_ content increases, indicating that the concentration of BECs that exist in different sites has increased as well. The broadening may be due to the Stark splitting of the energy sublevels induced by the crystal field on the outer shells of Bi^x^ ions, where the optical transitions occur, which are known for its high sensitivity to the local environment. In the case of the normalized spectra for λ_ex_ = 377 nm (Fig. 5e), four peaks at 970, 1040, 1094 and 1280 nm can be observed. The departure from the gaussian behavior of 970 nm peak is mainly due to the low quantum efficiency of our NIR-PMT detector below 940 nm. This peak was primarily considered due to the significant changes that we recorded with agreement to changes in Bi concentration (Fig. 5e) and GeO_2 _concentration, which will be covered in more detail hereafter. As Bi_2_O_3_ content increases from 0.1 to 0.4 mol %, the peak at 970 nm becomes less visible and the small peak at 1040 nm becomes dominant. This behavior can be either due to both the disappearance of BECs responsible for the 970 nm emission and increase of the ones responsible for 1040 nm, or due to the energy transfer taking place between these two BECs, in which one BEC grows at the expense of the decrease of the other one. In addition, under λ_ex_ = 377 nm, no peak shift has been observed for the 1280 nm peak with respect to the change in Bi_2_O_3_ content. This indicates that such radiative emission is from a BEC determined by some Bi^x^ ion where the transition probability is the same under any pumping wavelength. For all λ_ex_, the luminescence intensity shows a progressive increase as Bi content increases, until 0.3 mol %, while a decrease occurs beyond that value, for the sample 56Ge–0.4Bi. As previously discussed, the sample 56Ge–0.4Bi shown a noticeable larger degree of Bismuth reduction in comparison with the other samples (Fig. 1a).

Several authors reported a decrease in the NIR luminescent intensity as consequence of the excessive reduction of Bi^x^ species, in which *x* is the oxidation state of Bismuth that forms the BEC, to lower valence states in the glasses^43^. As melting temperature increases, Bi^x+^ is reduced according to the sequence: Bi^3+^ → Bi^2+^ → Bi^+^ → Bi → Bi clusters (such as Bi_2_, Bi^-^_2_, Bi_3_) → (Bi)_n_, where (Bi)_n_ represents Bi metallic colloids^44^. In particular, a reduction towards metallic Bi (Bi^o^) state and later nucleation and growth of Bi clusters was reported^43^. The formation of Bi clusters is considered detrimental since it limits the efficiency of optical fiber amplifiers^5^. In our work, the oxidation state of Bi^x^ ions starts at x = 3, since the precursor used is Bi_2_O_3_, and hence we may expect a reduction of the Bi^x^ ions during the fabrication process towards lower oxidation states and evolving to produce the dominant Bi species. In this regard, a diffusion limited glass matrix would help in creating more dominant species in the glass network, preferentially Bi^0^, and limit the formation of clusters or nanoparticles. In fact, in BDOFs, the amount of Bismuth precursor concentration used for doping is very low (about 0.02 at%) in order to reduce the clustering of Bi^0^ ions^5,14^.

In literature, there are plenty of theories about the possible origin of Bi^x^ luminescence for both BDGs and BDOFs. In BDOFs, either germanosilicate or pure silica, the NIR emission in the range of 1380–1530 nm is usually assigned to Si-BECs^18^. In contrast, the emission in the range of 1600–1800 nm is ascribed to Ge-BEC, and exists in both germanate^2^ and germanosilicate hosts^2,18^. Those BECs are thought to be formed by interstitial Bi^0^ linked to intrinsic defects of the glass, that is, Bi^0^ with ≡Si–Si≡ oxygen vacancies and Bi^0^ with ≡Ge–Ge≡ oxygen vacancies, respectively. For Bi^x^-doped bulk glasses, numerous studies suggested that the emission around 1260 nm, under excitation wavelengths of 470 and 808 nm, are due to optical transitions from ^2^D_3/2_ → ^4^S_3/2_ of Bi^0^ state^19,45,46^. However, according to selection rules of quantum mechanics, this transition can be considered as a forbidden transition. On other hand, some works consider the NIR emission of Bi^x^ is due to the transition happening between the ^3^P_1_ → ^3^P_0_ of Bi^+^ ion^17,47^. In our current work, the peak position and FWHM is different from each other for the three excitation wavelengths, which suggests that BECs could be formed by Bi^x^ in lower valence state, Bi^0^ ion according to the most agreed theories in literature, and the transition nature relies on the vicinity of atoms or local environment for the involved Bi^x^ ion. Therefore, the atoms that surround the Bi ion, such as Si, Ge, Al or Ba for our glass system, determine the spin–orbit interaction and the crystal field (Eq. 1), and modify in a different way the emission spectrum features, even for the same Bi ion. To unveil the possible oxidation state of the involved Bi^x^ ions and thus the origin of these emissions, different electronic transitions for Bi^2+^, Bi^1+^ and Bi^0^, identified in^48^, are shown in Table 2. Furthermore, in order to know which of the electronic transitions are allowed, ΔS = 0, or forbidden, ΔS ≠ 0, as well as its nature (ED—electric dipole, MD—magnetic dipole, EQ—electric quadrupole and MQ—magnetic quadrupole) for these Bi^x^ ions, we consider the selection rules for each possible electronic transition and its corresponding emission wavelength. It is important to highlight that “forbidden” transitions are still possible, and sometimes observable, but they are less intense since occur less frequently than allowed transitions when the molecules are exposed to electromagnetic radiation (See Table 2).

**Table 2:** Nature of optical transitions based on selection rule criteria for different Bi^x^ ions.

Bi^x^ ion	Electronic transitions	ΔS	Emission line of isolated Bi^x^ ion (nm)*	Observed emission peak (nm)**	Transition type	Splitting (= $\frac{2J + 1}{2}$)
Bi^2+^	^1^S_0_ → all	− 1/2	–	–	FT	–
	^2^S_1/2_ → ^2^P_3/2_	0	135	–	EQ	–
	^2^S_1/2_ → ^2^P_1/2_	0	105	–	EQ	–
	^2^P_3/2_ → ^2^P_1/2_	0	481	–	EQ	–
Bi^1+^	^1^S_0_ → ^1^D_2_	0	976	–	EQ	^–^
	^1^S_0_ → ^3^P_2_	1	368	–	FT	^–^
	^1^S_0_ → ^3^P_1_	1	324	–	FT	–
	^1^S_0_ → ^3^P_0_	1	226	–	FT	–
	^1^D_2_ → ^3^P_2_	1	591	–	FT	–
	^1^D_2_ → ^3^P_1_	1	485	–	FT	–
	^1^D_2_ → ^3^P_0_	1	295	–	FT	–
	^3^P_2_ → ^3^P_1_	0	2698	–	MD	–
	^3^P_2_ → ^3^P_0_	0	587	–	EQ	–
	^3^P_1_ → ^3^P_0_	0	751	–	MD	–
Bi^0^	^2^P_3/2_ → ^2^P_1/2_	0	869	N.M	MD	–
	^2^P_3/2_ → ^2^D_5/2_	0	550	N.M	EQ	–
	^2^P_3/2_ → ^2^D_3/2_	0	460	N.M	ED	–
	^2^P_3/2_ → ^4^S_3/2_	1	301	N.M	FT	–
	^2^P_1/2_ → ^2^D_5/2_	0	1607 (ΔE = 21,661–15,437 cm^−1^)^#^	1280/1320	EQ	^2^P_1/2_ (1) ^2^D_5/2_ (3)
	^2^P_1/2_ → ^2^D_3/2_	0	976 (ΔE = 21,661–11,419 cm^−1^)^#^	975/1050/1080	EQ	^2^P_1/2_ (1) ^2^D_3/2_ (2)
	^2^P_1/2_ → ^4^S_3/2_	1	462	N.M	FT	–
	^2^D_5/2_ → ^2^D_3/2_	0	2489	N.M	MD	–
	^2^D_5/2_ → ^4^S_3/2_	1	876	N.M	FT	–
	^2^D_3/2_ → ^4^S_3/2_	1	876	N.M	FT	–

*Those emission wavelengths are assigned to an isolated Bismuth ion^48^; # it indicates values reported in^48^; **Observed emission peak in our current work (extracted from Fig. 5). Manifold due to splitting is indicated in parenthesis in splitting column. The following nomenclature is used: N.M.—not measured.

**Figure 6:**
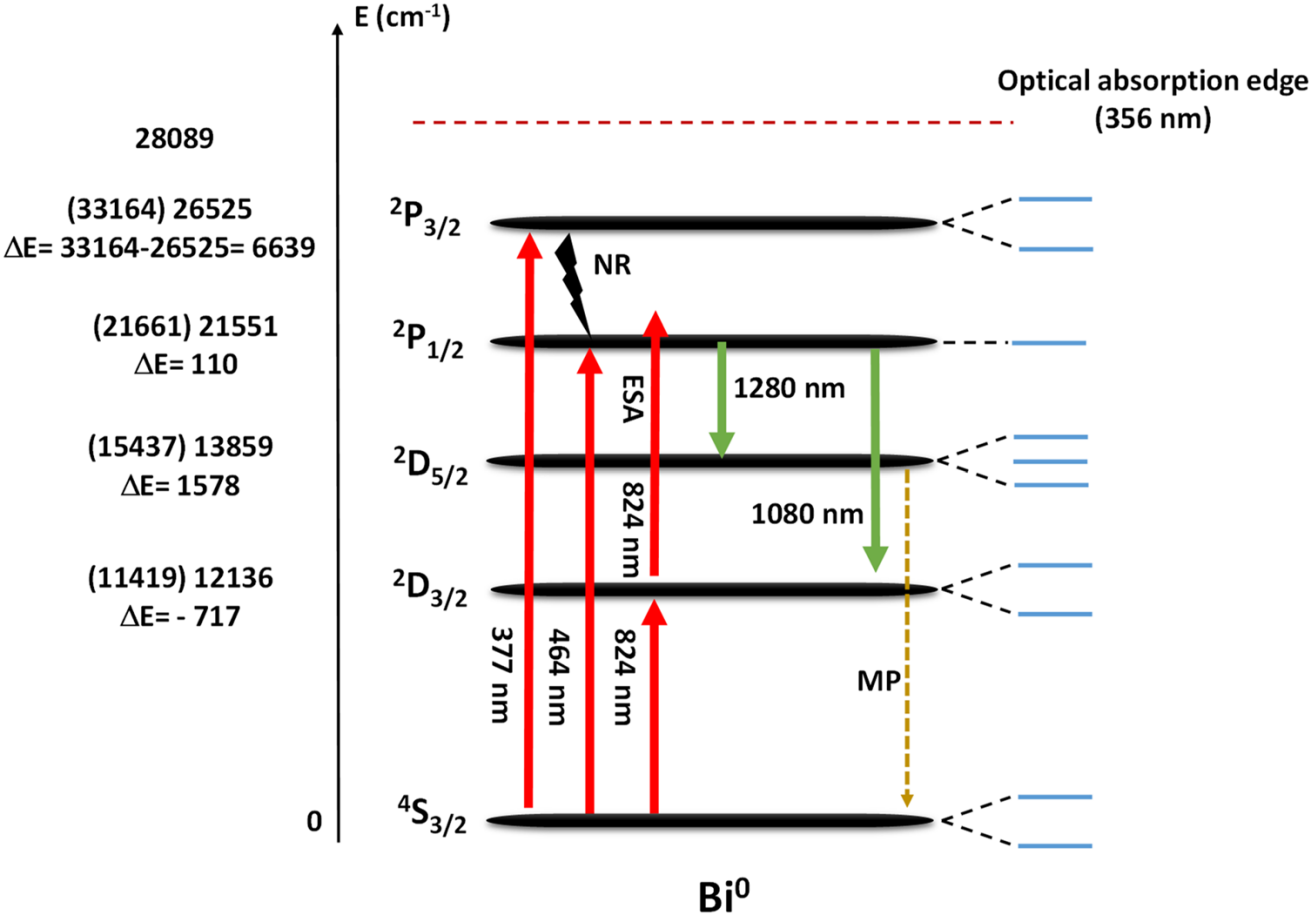
Energy level scheme for Bi^0^ in the developed Bi^x^-doped germanosilicate glasses. Energy values included in parenthesis are extracted from^48^ while the values listed on the energy axes are extracted from the maximum peaks of luminescence spectra in Fig. 5. The optical absorption edge is determined from the absorption spectrum (Fig. S2). In addition, on the right side, it is shown the manifold splitting of each energy level. Following abbreviations are used: NR—non-radiative decay, MP—multiphonon decay, and ESA—excited state absorption.

From Table 2, we can see that most of the transition probabilities for Bi^2+^ and Bi^1+^ ions are forbidden, and the only allowed ones are in the UV or VIS region. Considering Table 2 and our results for NIR region (Fig. 5), we can state that neither Bi^2+^ nor Bi^1+^ ions are present in our glass system. Additionally, in the absorption spectra of our glass samples provided in Fig. S2 of supporting information, we could not trace any absorption band related to Bi^1+^, such as the transitions ^3^P_0_ → ^3^P_1_ (~ 751 nm) or ^3^P_0_ → ^3^P_2_ (~ 587 nm), nor related to Bi^2+^, such as ^2^P_1/2_ → ^2^P_3/2_ (~ 481 nm) transition, which reiterates the fact that in our samples the most probable optical transitions that occurring are due to the presence of Bi^0^ instead of other Bi^x^ ions species. Besides, for Bi^0^ ions, the ^2^P_1/2_ → ^2^D_5/2_ and ^2^P_1/2_ → ^2^D_3/2_ NIR electronic transitions are allowed, and both are EQ. Also, according to Eq. 1, when an ion is placed in a glass matrix, it is subjected to different interactions that affect their atomic energy level position/values and hence its radiative emission. The first two terms $H_{o}$ and $H_{C}$ have a negative sign, and then, the position of high energy levels can shift to a lower energy value (downshift) due to central and Coulomb field. Similarly, low energy levels can shift its position towards higher energy values (upshift) due to spin–orbit coupling and crystal field splitting, described by last two terms of the Hamiltonian in Eq. (1), $H_{{{\text{SO}}}}$ and $H_{{{\text{CF}}}}$. When Bi^x^ ions are introduced into a glass matrix, their energy levels are sensitive to this kind of upshifts and downshifts. We made a comparison to an isolated Bi ion, in which the ΔE values determined for our current work relating various types of shifts are shown in Fig. 6. The figure shows a schematic of the energy levels for Bi^0^ under the different pumping wavelengths employed here as well as the radiative transitions responsible for the NIR emission in our samples (see also Table 2). The optical absorption edge for the glasses was determined from the absorption spectra measured for sample 56Ge-0.2Bi as shown in Fig. S2. The energy values between parenthesis for each level are the ones reported in^48^, while the values assigned for the energy levels are experimentally obtained from the maximum peak of luminescence spectra recorded for our glasses (Fig. 5). Thus, a downshift is obtained for the ^2^P_3/2_, ^2^P_1/2_ and ^2^D_5/2_ levels, with values of ${\text{E}}$ = 6639, 110 and 1698 cm^-1^, respectively. Similarly, an upshift of ${\text{E}}$ =− 717 cm^−1^ (the negative sign indicates the upshift of the energy levels) occurs for the ^2^D_3/2_ level. Shifts lower than 2000 cm^−1^ are the result of spin–orbit interaction. On the other hand, a ${\text{E}}$ = 6639 cm^−1^ is due to multielectron repulsion since the ^2^P_3/2_ energy level is inside the conduction band of the glass. It is important to mention that the emission obtained under 824 nm pumping is probably due to an upconversion process due to excited state absorption (ESA), which is possible due to the non-linear characteristic of these samples (see Fig. 4).

In summary, the inhomogeneous broadening occurs in glassy materials in which the dopant ions are located in multiple sites, which results in the broadening of the emission linewidth^39,49^ and blueshift or redshift of the peak emission wavelength. The Stark splitting and inhomogeneous broadening depend on the amount of doping concentration in the glasses and increases as doping amount increases^50^. Particularly, in Bi^x^-doped glasses, there are works reporting that the emission width broadening is associated with the formation of various BECs in the glass^51^.

**Figure 7:**
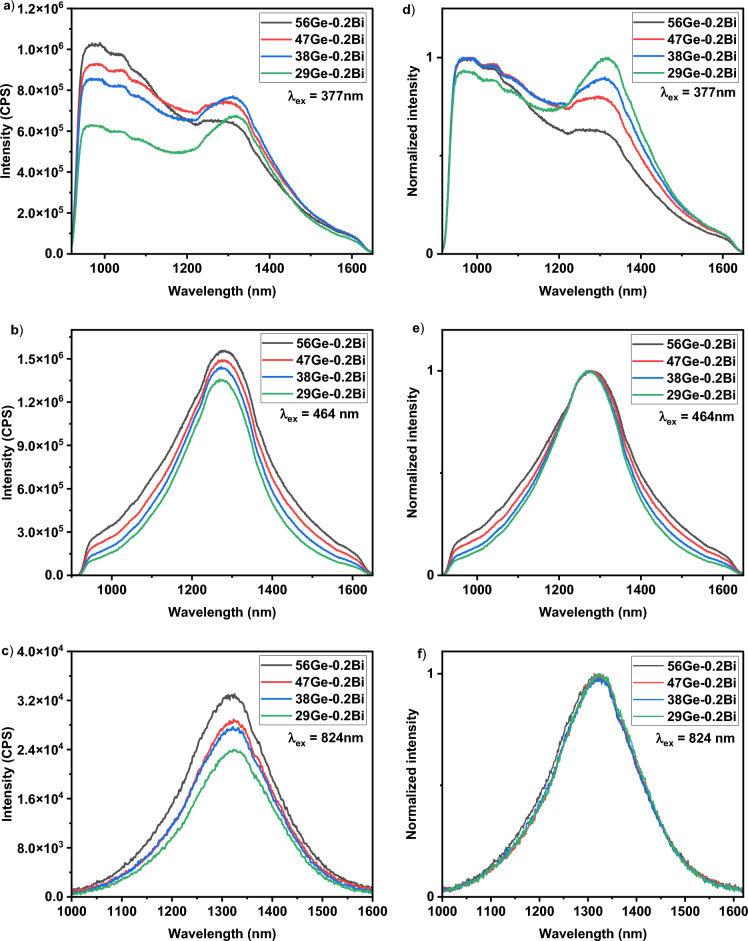
Luminescence spectra for glasses in the series (56 − x) Ge–0.2Bi, under the following excitation wavelengths: (**a**) 377 nm, (**b**) 464 nm, (**c**) 824 nm; The corresponding normalized spectra for excitation wavelengths of: (**d**) 377 nm, (**e**) 464 nm, (**f**) 824 nm.

With the aim of tailoring Bi^0^ NIR luminescence through the glass matrix, the samples in the series (56-x) Ge–0.2Bi were prepared. The luminescence spectra measured for those samples annealed at 620 °C for 5 h are depicted in Fig. 7. Similar contour plots of emission-excitation as in Fig. 5a were observed for (56-x) Ge–0.2Bi series, therefore the same λ_ex_ are also used for this glass series, that is, 377 nm (Fig. 7a,d), 464 nm (Fig. 7b,e) and 824 nm (Fig. 7c,f). Under λ_ex_ = 377 nm, an ultrabroadband emission in the range of 925–1630 nm was observed, with a FHWM of up to ~ 495 nm for the sample 29Ge–0.2Bi, and down to 443 nm for the sample 56Ge–0.2Bi (see Table 3). From the normalized spectra shown in Fig. 7d, the two previously discussed peaks at 980 and 1280 nm can be seen along with the two small shoulder peaks at 1040 and 1094 nm. According to literature, an emission centered at around 955 nm occurs in BDOFs, which it is assigned to Ge-BECs^18^. Based on this, the first peak in Fig. 7d can be assigned to Ge-BEC, which agrees with the fact that its intensity decreases as Ge content decreases. However, the emission usually ascribed to Si-BEC (1360–1530 nm) and other related Ge-BECs responsible of L- and U-band emissions, were not observed in our glasses. Moreover, the emissions that we observed, peaked at 1280 nm for λ_ex_ = 377 nm, 1270 nm for λ_ex_ = 464 nm and 1320 nm for λ_ex_ = 824 nm, were not reported in literature, to date, for the works dealing with BDOFs. No peak shift, with the change in GeO_2_ content, was exhibited by the peaks at 970, 1040, 1094 and 1280 nm peak. The fact that the peak at 1280 nm becomes more dominant and its intensity increases as SiO_2_ content increases under λ_ex_ = 377 nm, could initially lead to think that this BEC is more dependent on Si rather than Ge. However, the possibility of energy transfer between the BECs, with the same or different symmetry, located at 970, 1040 and 1090 to 1280 nm, cannot be neglected. A possible explanation for the presence of these BEC-Ge and BEC-Si might be that Germania and Silica have the same tetrahedral structure, and therefore the nature of the environment for Bi^0^ ion is the same. Regarding emissions at 1270, 1280 or 1320 nm observed in our work, all can be assigned to electronic transition ^2^P_1/2_ → ^2^D_5/2_ of the Bi^0^ ion (Table 2). The different positions of the peak maximum, that is the blue/redshift regarding calculated value for Bi^0^ (Table 2), are due to the spin–orbit interaction. Furthermore, the observed broadbands are due to the crystalline field effect around Bi^0^ ion, and thus, the presence of different sites in the glass network results in an inhomogeneous broadening.

**Table 3:** FWHM values for the fabricated samples in the series (56 − x) Ge–0.2Bi.

λ_ex_ (nm)	FWHM (nm)			
	56Ge–0.2Bi	47Ge–0.2Bi	38Ge–0.2Bi	29Ge–0.2Bi
377	443	465	483	494
464	274	246	229	218
824	204	199	198	196

Under λ_ex_ = 464 nm, the FWHM of NIR emission peaked at ~ 1270 nm, can be tuned from 218 nm for 29Ge–0.2Bi glass sample to 275 nm for the sample 56Ge–0.2Bi, as shown in Fig. 7b and given in Table 3. In this case, the luminescence intensity shown an increase as GeO_2_ content increases (Fig. 7b,e). This may be primarily due to the decrease in the phonon energy with increase in GeO_2_ content and vice versa for SiO_2_ which led to non-radiative emission in the samples. As it is noticeable from the Raman analysis (Fig. 3), the growth of peaks at 840 cm^−1^ and 1050 cm^−1^ suggests an enhancement in vibration modes of Si–O–Si bonds with higher SiO_2_ concentration. Such non-radiative emission is due to a multiphonon decay, and it is more probable than an EQ transition (see Table 2 and Fig. 6). The normalized luminescence spectra depicted in Fig. 7e shows the absence of any shift in the emission maximum when Ge content is modified in the glass matrix. For λ_ex_ = 840 nm a similar trend is observed for the NIR emission peaked at 1320 nm (See Fig. 7c,f). However, the FWHM showed a more subtle difference with the increase of Ge concentration (See Table 3). For both λ_ex_ = 464 and 824 nm, a decrease of the FWHM as Ge content decreases was noticed (Table 3), opposite to what is observed in the case of λ_ex_ = 377 nm. The rise in emission width with decreasing Ge content in the case of λ_ex_ = 377 nm, might be due to an increase of energy transfer processes that occur between several BECs. On the other hand, the Z of Ge is larger than Si, and hence, the expected energy value of the final three terms of the Hamiltonian included in Eq. 1 is larger for Ge in comparison with Si. This suggests that the impact of several Hamiltonian interactions can manipulate the emission width and hence the FWHM shown an increase for λ_ex_ = 464 and 824 nm with increase in Ge content, as experimentally observed (Fig. 7e,f). Furthermore, the energy transfer that occurs between different BECs may be a less probable process in the case of these λ_ex_ = 464 and 824 nm.

#### Lifetime measurements

**Figure 8:**
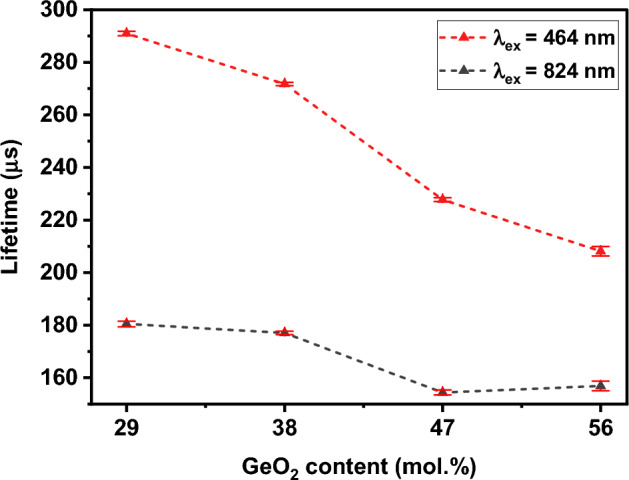
Lifetime measured for the glasses in the series (56-x) Ge-0.2Bi for 1270 nm peak, under λ_ex_ = 464 nm, and for 1320 nm peak, under λ_ex_ = 824 nm (error bars are included).

Figure 8 depicts the luminescent lifetime values recorded for the samples in the series (56-x) Ge–0.2Bi, for the emission at 1270 nm when excited using λ_ex_ = 464 nm and for the emission at 1320 nm under λ_ex_ = 824 nm. All the decay curves were fitted with single exponential decay equation and are displayed in Figs. S3 and S4 of supporting information section. A maximum value of 290 µs for the sample 29Ge–0.2Bi and a minimum value of 208 µs for sample 56Ge–0.2Bi were measured under λ_ex_ = 464 nm, while under λ_ex_ = 824 nm, the maximum and minimum values recorded were 180 µs and 156 µs for the samples 29Ge–0.2Bi and 56Ge–0.2Bi, respectively. Overall, the lifetime values tend to decrease as GeO_2_ content increases, which is an opposite behavior to the one observed for the luminescence intensity. This might be the result of an increased interaction between different BECs in the glass network^43^, since there is a possibility that more BECs were created in the glass network as the concentration of GeO_2_ increased, which would explain the reason of the observed rise in luminescence intensity with the increase of Ge content (Fig. 7). Furthermore, the range of lifetime values observed under both excitations is different from each other, which might be due to that the BEC responsible for λ_ex_ = 464 nm is directly excited and it is different from the one excited under λ_ex_ = 824 nm, via upconversion. In this context, the Bi^0^ exists in different local environments or sites and therefore, the probability of population inversion under different mechanisms is different, such as ground state absorption (GSA) and ESA.

## Conclusion

We fabricated Bi^x^-doped germanosilicate glasses, on the system SiO_2_–GeO_2_–BaO–Al_2_O_3_, with ultrabroadband NIR photoluminescence emission in the range of 925–1630 nm, that can be tuned through excitation wavelength, host glass composition and Bi dopant concentration. Raman and refractive index measurements showed that as GeO_2_ content decreases, SiO_2_ replaces it in the glass structure, and consequently more Si–O–Si bonds emerge in the glasses, connected through BOs. This results in an increased thermal stability and phonon energy. The addition of GeO_2_ enhances the intensity and FWHM of the ultrabroadband NIR luminescence in the glasses, attaining up to ~ 490 nm bandwidth for λ_ex_ = 377 nm. It is worth highlighting that the amount of Bi_2_O_3_ selected for the whole study is in the range of 0.1–0.4 mol.%, considerably lower than the Bi concentration considered in most of research works reported to date in literature for Bi^x^-doped bulk glasses ($\ge$ 1 mol%). In the light of our experimental and theoretical findings, we attribute the origin of Bi^x^ NIR luminescence observed under different λ_ex_s to Bi^0^. Moreover, it is included an explanation for the observed phenomena that affect the NIR luminescence of Bi^x^, such as the observed inhomogeneous broadening, stark splitting and blueshift/redshift of emission peaks. The present study provides novel insights into the impact of changing composition, excitation wavelength and Bi concentration in Bi^x^-doped germanosilicate glasses, which will be helpful for designing future Bi^x^-doped optical fiber amplifiers for the O-, E-, S-, C- and L- telecommunication bands with improved performance.

### Supplementary Information

Supplementary Information.

## Data Availability

The datasets generated during and/or analysed during the current study are available from the corresponding author on reasonable request.
