# Supplementary material for: Tailoring ultrabroadband near‐infrared luminescence in Bi-doped germanosilicate glasses
Source: Sci Rep. 2023 Dec 21;13:22852. doi: 10.1038/s41598-023-49898-1 (PMC10739908; doi:10.1038/s41598-023-49898-1)
Supplement: Supplementary file 1 — Supplementary Information. [file 41598_2023_49898_MOESM1_ESM.docx]

**Supporting information**

**Tailoring ultrabroadband near‐Infrared luminescence in Bi-doped germanosilicate glasses**

**A.Mehaboob*, V. Fuertes, V.A.G. Rivera, Y. Messaddeq**

Centre d’optique, Photonique et Laser, 2375 Rue de la Terrasse, Université Laval, Québec, QC, G1V 0A6, Canada

*Corresponding Author: [anees-mehaboob.cheriyathu-valappil.1@ulaval.ca](mailto:anees-mehaboob.cheriyathu-valappil.1@ulaval.ca)

**Fig S1:** a) Raman spectra recorded for the glasses in the series 56Ge-xBi and b) normalized Raman spectra measured for the same series.

**Fig S2:** Optical absorption spectra for the glasses in the series 56Ge-xBi. The optical absorption edge calculated from the absorption spectrum for the sample 56Ge-0.2Bi is approximately 356 nm. No absorption bands related to Bi^x^ were observed for all the glasses.

**Fig S3:** Luminescent decay curves recorded for the samples in the series (56-x) Ge-0.2Bi when excited under λ_ex_ = 464 nm at 1270 nm. The lifetime values were given in the inset of the graphs.

**Fig S4:** Luminescent decay curves measured for the samples in the series (56-x) Ge-0.2Bi under λ_ex_ = 824 nm at 1320 nm. The lifetime values calculated were provided in the inset.

| **Sample** | **T_g_ (**^0^C**)** | **T_x_ (**^0^C**)** | **ΔT (**^0^C**)** |
| --- | --- | --- | --- |
| 56Ge-0.2Bi | 635 | 746 | 111 |
| 47Ge-0.2Bi | 641 | 817 | 176 |
| 38Ge-0.2Bi | 643 | -- | -- |
| 29Ge-0.2Bi | -- | -- | -- |

**Table S1:** The characteristic temperatures and DSC parameters calculated for the glasses in the series (56-x) Ge-0.2bi.
